# Developing a model for predicting safety performance of nurses based on psychosocial safety climate and role of job demands and resources, job satisfaction, and emotional exhaustion as mediators

**DOI:** 10.1186/s40359-023-01223-1

**Published:** 2023-06-22

**Authors:** Fatemeh Abdi, Mehdi Jahangiri, Mojtaba Kamalinia, Rosanna Cousins, Hamidreza Mokarami

**Affiliations:** 1 Department of Occupational Health and Safety Engineering, School of Health, Shiraz University of Medical Sciences, Shiraz, Iran; 2 Department of Psychology, Liverpool Hope University, Liverpool, UK; 3 Department of Ergonomics, School of Health, Shiraz University of Medical Sciences, PO Box 71645-111, Shiraz, Iran

**Keywords:** Safety performance, Work-related stressors, Psychosocial safety climate, Healthcare workers, Nurses, Structural equation modeling

## Abstract

**Background:**

The present study aimed to develop a model for predicting the safety performance of nurses based on psychosocial safety climate (PSC) and the role of job demands and resources, job satisfaction, and emotional exhaustion as mediators.

**Methods:**

A cross-sectional study using structural equation modeling (SEM) was carried out among nurses in Iran. Data were collected using the Psychosocial Safety Climate questionnaire, Neal and Griffin’s Safety Performance Scale, the Management Standards Indicator Tool, the Effort-Reward Imbalance questionnaire, the Michigan Organizational Assessment Job Satisfaction subscale and the Maslach Burnout Inventory.

**Results:**

Surveys were distributed to 340 nurses provided informed consent. After removing incplete surveys, data from 280 partipants were analysed. The completion rate was 82.35%. The SEM results indicated that PSC can directly and indirectly predict nurses’ safety performance. The final model showed an acceptable goodness of fit (*p* = 0.023). It indicated that PSC, job demands, and job satisfaction were directly related to safety performance, and also that PSC, emotional exhaustion, job resources, and job demands were all indirectly related to safety performance. Also, PSC had a significant relationship with all mediator variables, and job demands had direct effect on emotional exhaustion.

**Conclusions:**

The current study presented a new model for predicting safety performance in nurses in which PSC, both directly and indirectly, plays an important role. In addition to paying attention to the physical aspects of the workplace, healthcare organizations should also take into account PSC to improve safety. Next steps in reducing safety issues in nursing is to develop intervention studies using this new evidence-based model as a framework.

## Introduction

Nurses working in hospital and medical settings face various physical, chemical, and psychosocial work-related risk factors. These include patient care and relocation, exposure to blood pathogens and infectious diseases, needlestick and sharps injuries, poor workstation design, violence, high levels of stress, high workload, and years of shift work [1–5]. In addition to the negative effects on their health, these risk factors can engage medical staff in unsafe performance and lead to human errors which ultimately impacts on patient safety [5–7]. There is agreement that knowledge, skills, and judgments associated with safe and appropriate performance is a requirement for safe practice in nursing [8], and similarly the general definition of safety performance as being behaviors that employees exhibit in the workplace adhere to and promote safety [9] is pertinent in the context of nursing [10]. Therefore, it is necessary to identify and evaluate the factors affecting the occurrence of these risk factors in clinical and hospital settings and, consequently, take targeted preventive measures [11, 12]. There are several gaps in the literature regarding predicting safety performance of nurses – particularly with respect to psychosocial climate and the impact of this on safe and unsafe behaviors.

Unsafe behavior is one of the important factors leading to occupational accidents and human errors [11]. Early research on unsafe behaviors tended to measured safety performance based on number of notified incidents or safety events. It is now appreciated that this was an unsatisfactory approach as it provided overestimates of safety performance and typically did not take into account psychological antecedents that predict safety performance [13]. Griffin and Neal [14] defined safety performance as an aspect of work performance. Following the job performance literature, they conceptualized safety performance as individual work behavior based on two particular behaviors: safety compliance and safety participation [11, 15], and implicated the involvement of psychological antecedents. A subsequent longitudinal study by Neal & Griffin [16] provided evidence that safety performance has a lag effect on future safety incidents. This supports the involvement of psychosocial antecedents and the recent use of safety performance as a criterion variable [13].

Safety climate is an important construct for understanding the way work safety is collectively perceived by employees [17]. That is, safety climate perceptions are cognitive in nature, and provide a frame of reference for work behaviors [18]. Moreover, it has been proposed that safety climate is an important factor that can affect occupational accidents and injuries through safety performance [19]. The relationship between safety climate and safety performance has been investigated as an upstream factor for safety performance. However, the conclusion from Griffin and Curcuruto’s [18] review of safety climate in organizations is that there are still many unknowns in the understanding of safety climate. Moreover, it has been suggested that one of the problems in examining this variable is that it deals only with the physical work-related factors and ignores the psychosocial work-related factors [20]. This is a challenge for developing models of predictors of safety performance as psychosocial risk factors are considered as important predictors of safe behaviors and work accidents [20, 21]. Dollard and Karasek [22] introduced a new construct: psychosocial safety climate (PSC) – originally to reduce work-related stress, and improve productivity. Dollard and Bakker [23] similar elaborated upon how PSC can protect worker health & safety, and PSC can be used as an upstream factor to predict employees’ safety performance [23]. This has been endorsed in subsequent studies [21, 24]. PSC refers to policies, practices, and procedures to protect mental health and safety in employees. PSC mechanism affects other psychosocial risk factors, and a multi-stage process in the process of psychosocial and physical injuries and helps provide an environment with safe behaviors [21, 24].

Based on its original conception [22] the job demands-resources model [25] is used for a better understanding of the PSC impact process. In this model, job demands, although not necessarily a negative factor, are by definition aspects of the job that require sustained physical or psychological effort and from this it can be deduced have certain physiological or psychological costs. Excessive demands can lead to unsafe behavior, which in turn poses a serious threat to medical staff and patient health [15]. Job resources refer to physical, social or organizational aspects of the job that can help a person achieve occupational goals and reduce job demands and related physiological and psychological costs, as well as initiate a motivational process for the person [15]. Examples of job resources include social support, job control and various forms of rewards [26].

Job satisfaction is one of the perceptual variables that can play an important mediating role in the relationship between job demands and job resources with safety performance [27]. Employees with high job satisfaction perform more safely, but employees who perform safely do not necessarily have a higher level of job satisfaction [28]. Emotional exhaustion is another perceptual variable that can mediate the relationship between job demands and job resources with safety performance [29–31]. Emotional exhaustion occurs in the long run following a decrease in employee job satisfaction [31]. A person suffering from emotional exhaustion does not have enough energy to face another working day or handle clients. Emotional exhaustion is affected by workplace characteristics [32, 33].

Following these arguments and despite the emerging evidence of importance of the PSC in predicting employees’ safety performance in frontline healthcare [34], there is a dearth of studies conducted in Iran that focused on the relationship between PSC and safety performance. Given that there is a procedural relationship between the PSC and employees’ safety performance, it is also necessary to identify and evaluate important factors mediating this relationship as a preliminary step to planning of control measures to ameliorate potentials for occupational accidents and promote employees’ mental health. Such planning requires extensive knowledge about influential factors and how they work, which is gained through modeling and simultaneous analysis of various variables affecting safety performance.

**Fig. 1:**
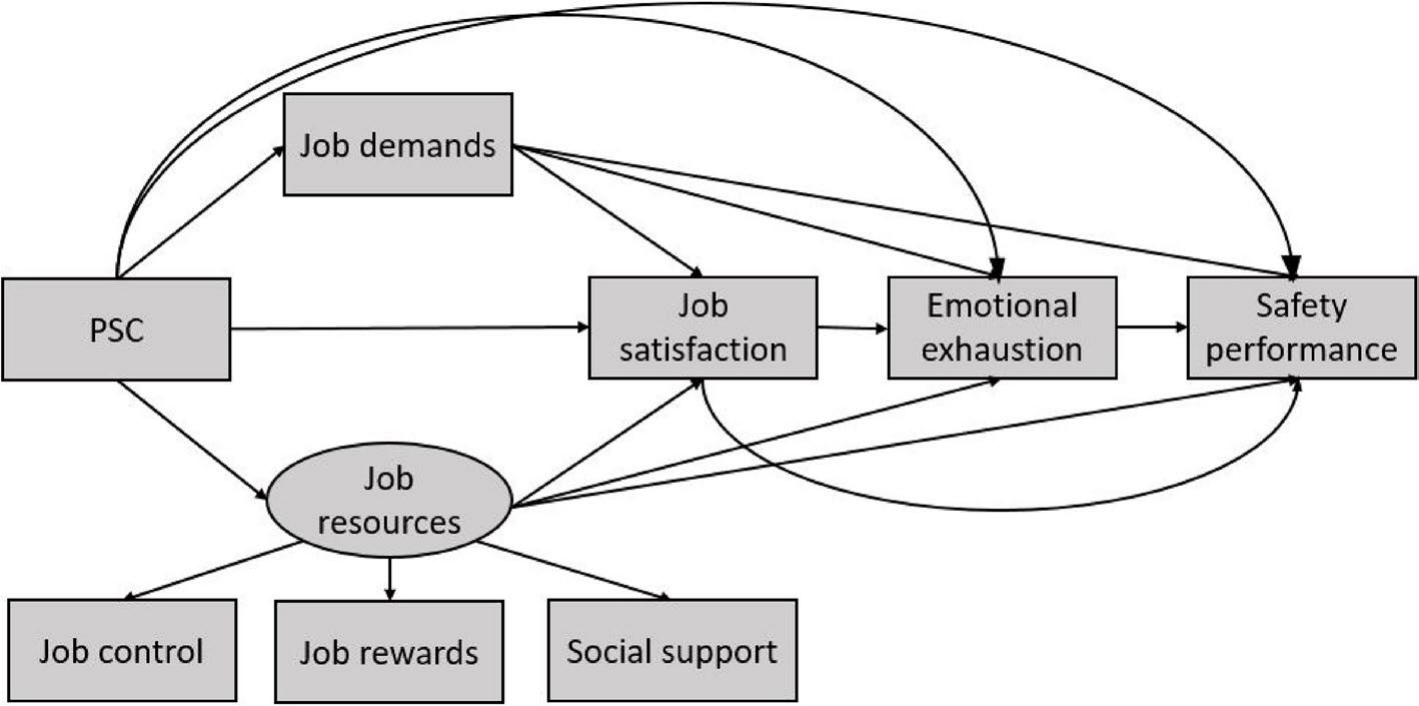
Hypothesized model of safety performance

Accordingly, using structural equation modeling (SEM), the aim of the present study was to explore the effect of the PSC on nurses’ safety performance, and to develop a conceptual model based on the potential mediating role of job demands and resources, job satisfaction, and emotional exhaustion in the relationship between PSC and safety performance: the most important organizational and occupational factors affecting nurses’ safety performance identified by our review of the literature. Critically, PSC has an important role in safety performance [21], and there are indications that PSC can affect safety performance through other psychosocial risk factors [24]. Thus, as shown in Fig. 1, we hypothesized that PSC would predict safety performance both directly, and indirectly though associations with other variables known as predictors of safety performance.

Specifically, we modelled PSC as a predictor variable, and hypothesized that job resources, entered as a latent variable made up of measures of job control, job rewards and social support [26], and job demands would be two other variables that can provide an explanation of the effect of PSC on various psychological factors, and ultimately safety performance. Other psychosocial risk factors specifically considered here are job satisfaction and emotional exhaustion as they are both important and common risk factors among healthcare workers and, as discussed, play a major role in safety performance and occupational accidents. On the other hand, these factors can be affected by PSC, job demands, and job resources. Therefore, job demands, job resources, job satisfaction, and emotional exhaustion were included in the conceptual model of the present study as mediating factors in the relationship between PSC and safety performance. To summarize: In this study, PSC was included in the hypothesized model as a predictor variable; job demands, job resources, job satisfaction, and emotional exhaustion were added to the model as mediating variables; nurses’ safety performance was considered as an outcome variable in the model. This study sought to test the relationships in the hypothesized model.

## Methods

### Study design and participants

The study used a cross-sectional-analytical survey design. First, we recruited a sample of experts to pre-test and evaluate the validity of the researcher’s translation of the Psychosocial Safety Climate (PSC-12) scale [24] that was used to collect information on employee safety performance. A targeted invitation to join the study in this capacity was sent to 30 nurses with experience of frontline nursing who were then working in administration, and 10 university professors (ergonomics, occupational health, and health promotion). All those invited agreed to take part and provided informed consent. All were volunteers, and no payment was made.

Recruitment to the main survey took place in the thirteen hospitals of the large metropolis of Shiraz, in south Iran affiliated to the Shiraz University of Medical Sciences. The inclusion criteria were being employed as a qualified nurse with at least one year of post-qualification service and working in frontline services. The exclusion criterion was working in an administrative role. The study was promoted widely in the hospitals using notices which invited all experienced frontline nurses in the city to attend one of three presentations about the study for more information and formal recruitment. The presentations were held at the University, at different times to cover all shift patterns. Members of the research team provided oral information about the study and its objectives, and there was an opportunity for potential participants to ask questions. To encourage supportive participation and collect sufficient data to represent the nurse population, at the request of the nurses, it was agreed that the demographic variables related to identity (wards, hospital, etc.) would be removed from survey at the data collection point, to ensure their anonymity. To further ensure confidentiality and anonymity, the potential participants were shown, how along with the survey, they would be provided with an unmarked sealed envelope to enter their survey and return directly to the researchers after completion. No payment was made to any participant.

A total of 340 nurses from the three presentation sessions agreed to join the study and all signed a written informed consent form. The survey was then distributed to each of the 340 participants, and later returned anonymously in the unmarked envelope provided. This sample size was considered sufficient to influence the detection of significant relationships necessary to test the hypothesized model based on tables for minimum returned sample size for continuous data [35] which indicates a minimum sample of 119 for a population of 10,000 when the alpha level for significance at set at 0.05 a priori, and 179 when following Salkind’s recommendation to oversample by as much as 50% when distributing questionnaires, to build in an anticipated non-completion rate [36].

### Measures

The survey comprised a brief demographic section, and the measures listed below.

#### Psychosocial safety climate (PSC-12)

The PSC-12 scale was used to collect information on employee safety performance [24]. It comprises three items in each of four pertinent areas: management commitment, management priority, organizational communication and organizational participation. Responses are on a five-point agreement Likert scale. Scores ranges from 12 to 60, and high scores are associated with a positive psychosocial safety climate.

Afsharian et al. [37] translated the PSC-12 scale into Persian for a cross-cultural study of PSC, and reported good reliability of their measure. This scale, however, was not available to us, therefore a fresh translation of the scale in English into Persian was undertaken based on the cross-cultural adaptation guidelines for instrument translation proposed by Beaton et al. [38]. Then, a sample of 30 nurses and 10 professors recruited as experts in the field were recruited to pre-test the questionnaire and to evaluate the face validity and qualitative content validity of our translation of the PSC-12. This group of nurses completed the questionnaire anonymously and then they were asked to evaluate the twelve scale items in terms of comprehensibility, wording, interpretations, cultural issues, and clarity. The participants’ feedback was used by the research team to make minor revisions to the items. The content validity of the scale was then assessed by measuring its content validity index (CVI) and content validity ratio (CVR). To determine CVI, the 10 professors were each asked to rate the relevance of each item. In accordance with good practice [39], the CVI should be greater than 0.79 is the item is suitable. To measure CVR, the professors rated the necessity of each item. According to Lawshe’s table’s [40] items with a CVR greater than 0.62 were valid. Structural analysis of the Persian PSC-12 was performed using confirmatory factor analysis (CFA) with the maximum likelihood estimation (MLE) method. The internal consistency of the scale was also assessed using Cronbach’s alpha index.

#### Safety performance

The validated Persian version of the 8-item Neal and Griffin’s Safety Performance Scale was used [41]. Kalteh et al.’s findings supported the two-dimensional structure (safety participation and safety compliance) associated with safety performance of the original version in English [42]. Items are measured using a 5-point Likert agreement scale. Scores range from 8 to 40. High scores indicate positive safety behaviors. Cronbach alpha was reported to be 0.90.

#### Job demands

The Demands subscale of the ‘Management Standards’ Indicator Tool [43] was used. This measure was developed to assess the perceived ability of employees to deal with the demands of their jobs. Each of the eight items is scored on a 5-point Likert Scale yielding scores between 8 and 40. Reliability was (Cronbach’s alpha = 0.89). The Indicator Tool was translated into Persian by Azad and Gholami [44]. They confirmed its validity and reliability for use in research in Persian.

#### Job resources

This latent variable was used to represent three observable aspects of work that provide help employees: job control, job rewards, and social support. Four validated subscales to used separately and to provide data. To measure Job control and Social support, three subscales of the aforementioned ‘Management Standards’ Indicator Tool [43, 44] were used: Control (6 items; Cronbach’s alpha = 0.78); Managerial Support (5 items; Cronbach’s alpha = 0.87), and Colleague Support (4 items; Cronbach’s alpha = 0.81). To measure Job rewards the Reward subscale (11 items) of Seigrist’s Effort-Reward Imbalance questionnaire [45]. This questionnaire was translated into Persian by Yadegarfar et al. [46] and its validity and reliability (Cronbach’s alpha = 0.85) were evaluated and confirmed. Items were scored on a 5-point Likert scale, including reverse coding where necessary to denote that high scores denote a high level of control, social support, rewards, and job resources.

#### Job satisfaction

The 3-item Job Satisfaction subscale (MOAQ-JSS) of the Michigan Organizational Assessment Questionnaire was used to measure employees’ job satisfaction [47]. This measure was translated into Persian by Mokarami [48] and they evaluated and confirmed its validity and reliability. Items were scored using a 6-point Likert scale. The second item was reversed to yield scores between 3 and 18, where a high score indicated higher job satisfaction.

#### Emotional exhaustion

The validated Persian version [49] of the 9-item emotional exhaustion scale of the Maslach Burnout Inventory (MBI) [50] was used. This scale measures the frequency in which employees experience feelings being emotionally drained by their work with using a 7-point response format ranging from ‘0 = never’ to ‘6 = every day’. A high score is indicative of higher emotional exhaustion.

### Statistical analyses

Kolmogorov–Smirnov tests were used to evaluate the normality of the data. Participants’ demographic characteristics (age, gender, education etc.) were analyzed using descriptive statistics including mean and standard deviation indices. To test the research hypotheses in the form of equations between variables, to consider the measurement error, to explain the relationships between the variables in the final model, and to eliminate competing models, more advanced analytical procedures were performed using structured equation modelling (SEM). All statistical tests were performed at the significance level of 0.05 (*p* < 0.05) using IBM SPSS Statistics for Windows, version 23 (IBM Corp., Armonk, N.Y., USA) and associated AMOS software. To evaluate the good fit of the SEM model, the root mean square error of approximation (RMSEA), the root mean square residual (RMR), the good fit index (GFI), the adjusted good fit index (AGFI), the comparative fit index (CFI), and chi-square to the degree of freedom ratio (χ^2^/df) were used. If the CFI value is 0.95 or higher, the RMSEA is less than 0.08, the GFI and AGFI values ​​are 0.8 or 0.9, and χ^2^/df is less than 3, the model fit is considered acceptable.

## Results

A total of 340 participants were recruited to the study. Sixty participants did not complete the survey instrument, and thus the data from 280 participants were used in the final analysis: A completion rate of 82.35%. The majority of the 280 nurses who participated in the study were female (79.3%), their mean age was 31.91 ± 6.68 years (range 21–54 years), and 57.5% were married whilst 42.5% were single. All nurses were employees, working full time, and the majority worked on the standard three-shift work-schedule (84.3%). Their average service records were 8.35 ± 6.71 years. The majority (95%) were educated to at least Bachelor’s degree level.

**Fig. 2:**
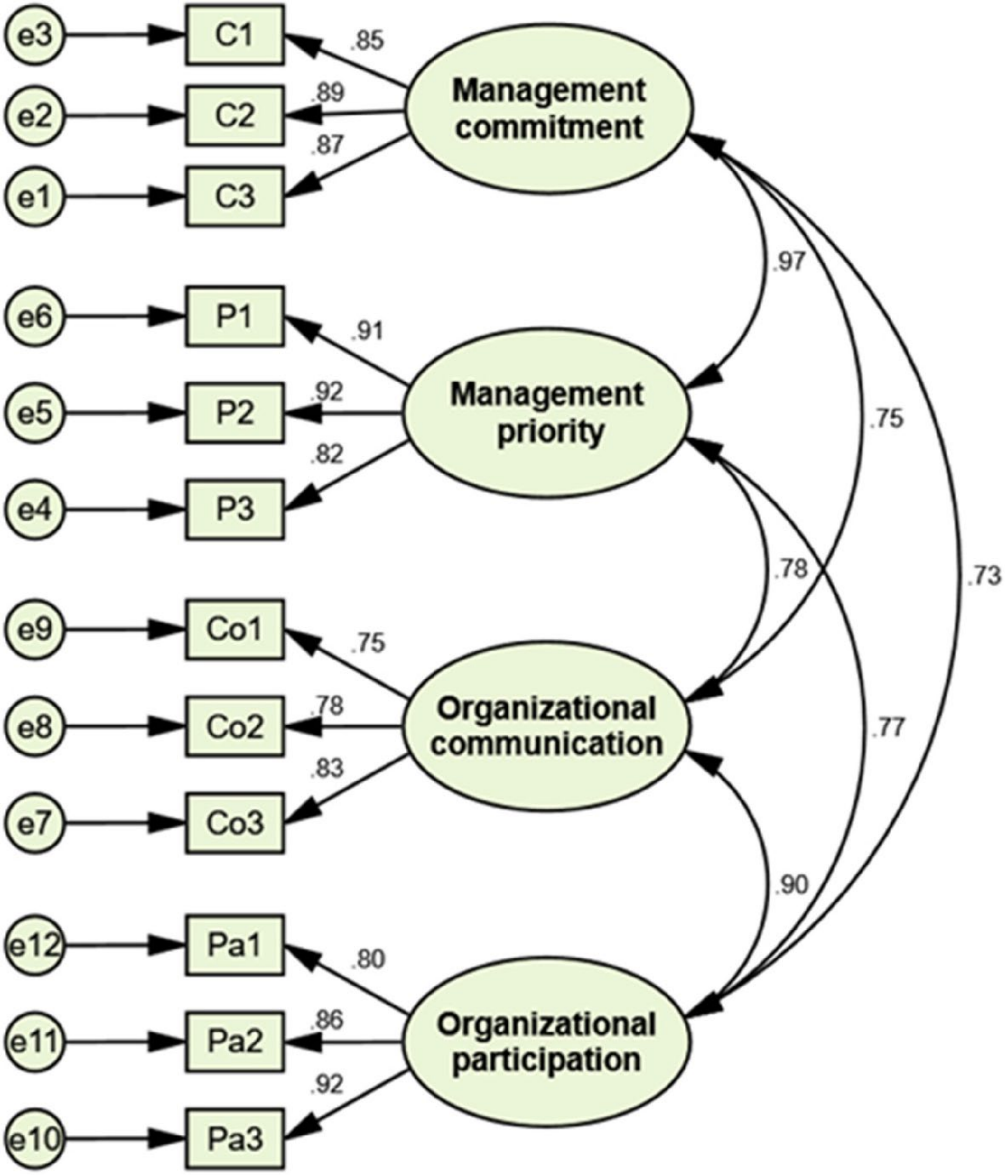
The four-factor model of the Persian version of Psychosocial Safety Climate Scale obtained by confirmatory factor analysis

Regarding the psychometric properties of the translated Persian PSC-12, the mean CVI score for the PSC-12 items was higher than 0.92, and the CVR score for all items was higher than 0.67, thus indicating good content validity. The path diagram of the CFA of the scale with standardized factor loadings of the items is shown in Fig. 2.

The goodness-of-fit indices were as follows: χ^2^ was 74.8, with 47 degrees of freedom (*df*); χ^2^/df = 1.58. RMSEA = 0.048, RMR = 0.044, GFI = 0.95, AGFI = 0.92, and CFI = 0.99. These indicators showed a very acceptable goodness-of-fit of the model. The factor loading values of the items measuring avoiding distraction and completing were in the range of 0.86 – 0.87, 0.80 – 0.91, 0.74 – 0.81 and 0.81 – 0.92 (*p* < 0.001), indicating the most desirable factor loading of the items in four dimensions of PSC-12.

The Cronbach’s alpha coefficient for PSC was 0.95 indicating excellent internal consistency. Similarly, the Cronbach’s alpha ‘if item deleted’ coefficients were in the range of 0.944 – 0.949, and the corrected item-total correlations were in the range of 0.68 – 0.84. The confirmatory factor analysis indicated that the original four-dimensional structure of the PSC-12 remained in the Persian translation (See Table 1).

**Table 1:** Construct validity of PSC-12 (*N* = 280)

Item	Mean (SD)	Corrected item–total correlations	Cronbach's Alpha if Item Deleted	Confirmatory Factor Analysis					
				**Standardized Regression Weight**				**Critical Rate**	***p***
Q1	2.58 (1.297)	.782	.946	.856				17.983	< .001
Q2	2.44 (1.298)	.783	.946	.877				18.772	< .001
Q3	2.44 (1.254)	.793	.946	.865					< .001
Q4	2.34 (1.249)	.838	.944		.904			17.395	< .001
Q5	2.40 (1.280)	.840	.944		.909			17.537	< .001
Q6	2.26 (1.261)	.775	.947		.804				< .001
Q7	2.60 (1.144)	.708	.949			.738		12.455	< .001
Q8	2.85 (1.223)	.683	.949			.795		13.695	< .001
Q9	2.62 (1.237)	.710	.949			.815			< .001
Q10	2.62 (1.240)	.717	.948				.808	17.179	< .001
Q11	2.29 (1.209)	.752	.947				.846	18.845	< .001
Q12	2.46 (1.242)	.798	.946				.919		< .001

Table 2 displays descriptive data regarding the research variables, their reliability, and the correlation coefficients between the variables. As can be seen, PSC was significantly correlated with all the research variables, and all the research variables, except control, had significant correlations with safety performance.

**Table 2:** Mean, SD, scale reliability and correlations between safety performance and independent variables (*N* = 280)

Variable	Mean	SD	α	1	2	3	4	5	6	7	8
1. 1. Safety Performance	32.12	5.2	.88								
2. Psychosocial Safety Climate	30.03	12.1	.95	.29^**^							
3. Emotional exhaustion	27.52	13.2	.92	-.28^**^	-.37^**^						
4. Job control	16.59	3.9	.68	.086	.32^**^	-.34^**^					
5. Colleague support	13.98	3.0	.79	.20^**^	.33^**^	-.27^**^	.39^**^				
6. Managerial support	17.18	4.7	.90	.23^**^	.33^**^	-.26^**^	.46^**^	.63^**^			
7. Job rewards	34.50	6.4	.71	.34^**^	.46^**^	-.52^**^	.40^**^	.49^**^	.60^**^		
8. Job satisfaction	12.58	3.3	.82	.27^**^	.33^**^	-.49^**^	.27^**^	.23^**^	.29^**^	.37^**^	
9. Job demands	25.31	5.40	.79	-.25^**^	-.32^**^	.53^**^	-.34^**^	-.18^**^	-.21^**^	-.48^**^	-.29^**^

^**^p < 0.01

**Fig. 3:**
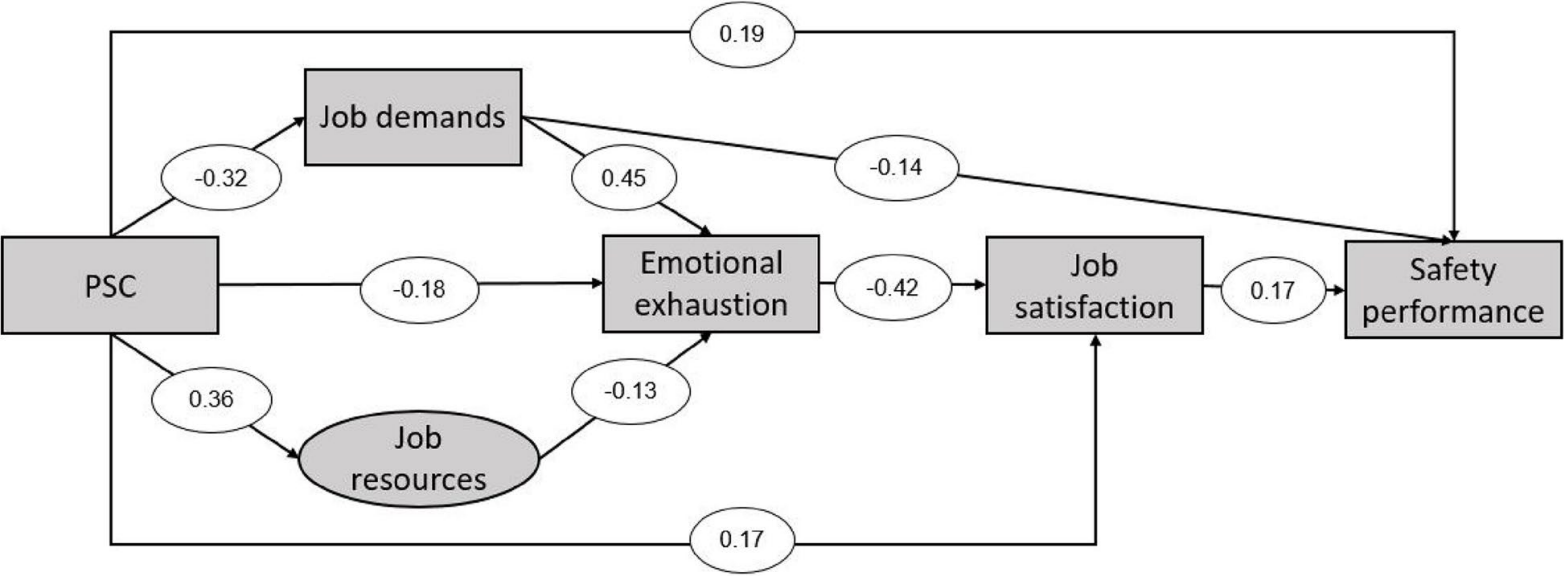
Final model with standardized path coefficients for mediating effects (*p* < 0.05)

The proposed model did not fit well with the data based on the theoretical framework of the study. Following the AMOS software guidelines, some modifications were made in the post-hoc model to develop a suitable model by removing or adding new paths (See Fig. 3).

The goodness-of-fit indices of the final model showed an acceptable goodness-of-fit of the model (*p* = 0.023), and were as follows: χ^2^/df = 2.60, GFI = 0.99, AGFI = 0.94, NFI = 0.96 and CFI = 0.97, TLI = 0.92, RMSEA = 0.076. These indicators showed a very acceptable goodness-of-fit of the model. The critical ratio (CR) and standardized coefficients of the final model paths are presented in Table 3.

**Table 3:** The critical ratio (CR) and standardized coefficients of the final model paths

Hypothesis	CR	Standardized Estimate (β)	*P*
PSC → Job demands	-5.69	-0.32	< 0.001
PSC → Job resources	6.54	0.36	< 0.001
Job resources → Emotional exhaustion	-2.42	-0.13	< 0.015
Job demands → Emotional exhaustion	8.71	0.45	< 0.001
PSC → Emotional exhaustion	-3.29	-0.18	< 0.001
Emotional exhaustion → Job satisfaction	-7.63	-0.42	< 0.001
PSC → Job satisfaction	3.05	0.17	< 0.002
Job satisfaction → Safety performance	2.76	0.17	< 0.006
PSC → Safety performance	3.13	0.19	< 0.002
Job demands → Safety performance	-2.40	-0.14	< 0.016

In the final model, PSC was directly involved in predicting safety performance (β = 0.19; *p* = 0.002). Furthermore, PSC had a significant relationship with all mediator variables in the model. A comparison of the mediating variables showed that only job demands (β = -0.14; *p* = 0.016) had a significant direct effect on safety performance. This variable had a relatively strong direct effect on emotional exhaustion (β = 0.45; *p* < 0.001). The latent variable job resources had only an insignificant direct effect on emotional exhaustion (β = -0.13; *p* = 0.015). Corrective indicators showed a new path to the effect of emotional exhaustion on job satisfaction. It should be noted that in the initial model, job satisfaction was considered a predictor of emotional exhaustion. Accordingly, emotional exhaustion had only a relatively strong significant direct effect on job satisfaction (β = -0.42; *p* < 0.001) and had no direct effect on safety performance. In the final model, job satisfaction was evaluated as the last variable mediating the relationship between PSC and safety performance. This variable had a significant effect on safety performance (β = 0.17; *p* = 0.006). The direct, indirect, and overall effects of all the studied paths in the final model are presented in Table 4. As shown in the table, PSC had significant indirect effects on emotional exhaustion (β = -0.19), job satisfaction (β = 0.17), and safety performance (β = 0.10). The beta coefficients for the overall effects of PSC, job resources, job demands, emotional exhaustion, and job satisfaction on safety performance were 0.291, 0.009, -0.175, -0.070, and 0.166, respectively.

**Table 4:** Direct effect, indirect effect, and total effect of outcome variables in the final model

Predictor variable	Outcome variable	Direct effect	Indirect effect	Total effect
**PSC**	Job resources	0.365	-	0.365
	Job demands	-0.322	-	-0.322
	Emotional exhaustion	-0.181	-0.192	-0.373
	Job satisfaction	0.169	0.158	0.327
	Safety performance	0.190	0.101	0.291
**Job resources**	Job resources	-	-	-
	Job demands	-	-	-
	Emotional exhaustion	-0.127	-	-0.127
	Job satisfaction	-	0.054	0.054
	Safety performance	-	0.009	0.009
**Job demands**	Job resources	-	-	-
	Job demands			
	Emotional exhaustion	0.450	-	0.450
	Job satisfaction	-	-0.190	-0.190
	Safety performance	-0.144	-0.031	175/0-
**Emotional exhaustion**	Job resources	-	-	-
	Job demands	-	-	-
	Emotional exhaustion			
	Job satisfaction	-0.423	-	-0.423
	Safety performance	-	-0.070	-0.070
**Job satisfaction**	Job resources	-	-	-
	Job demands	-	-	-
	Emotional exhaustion	-	-	-
	Job satisfaction			
	Safety performance	0.166	-	0.166

## Discussion

This study aimed to develop a model for predicting nurses’ safety performance behaviors based on psychosocial safety climate (PSC) using structural equation modelling to add to the extant literature. Overall, the results of the study showed that PSC can directly and indirectly predict the nurses’ safety performance. In addition to the direct effect of PSC on safety performance, PSC had a significant relationship with all the variables in the research model further illuminating understanding of safety performance of nurses. This study showed that PSC affected safety performance through job demands, job resources, emotional exhaustion, and job satisfaction. These were four mediating variables hypothesized to contribute to explaining the impact of PSC in predicting safety performance of nurses. All four mediators in the proposed model had a significant role in the final model. The hypothesized model was largely but not totally supported, as discussed below.

This study is the first to model important organization variables in the prediction of safety performance in nurses in Iran. It also adds to the global literature, as previously only a few studies have addressed the association between PSC and safety performance. Most available evidence supports the association of PSC with safety performance [19, 20, 51] – indeed using data from their cross-sectional study of US nurses, Manapragada et al. [19] concluded that nurses’ safety performance is continent on organizational management of psychosocial risks in their workplace. There is, however, one substantial longitudinal investigation using Australian healthcare staff that did not confirm a direct relationship between PSC and occupational accidents [34]. Nevertheless, in that study there could be underestimates of safety performance, as this was measured by reported accident rates [13]. This was acknowledged in the discussion of the findings.

The present study showed that if the PSC is high in an organization, then risks to employees’ mental health and safety behaviors are likely to be lower. When employees perceive that their safety and mental health are important to management it increases their positive emotions and motivation [18], and these emotions may have a positive effect on their safety behaviors. In addition, refering to the conservation of resources theory [52], it has been argued that in environments with high PSC levels, management actions to reduce stressors conserve employees’ energy and resources to spend them on improving safety behaviors [53]. Although this study did not specifically link their argument to nurses, including doctors and other health professionals, the fact that the central arguments have been endorsed in nurses, and in other service organizations [54] is a strong endorsement for a focus on seeking to provide high PSC to promote safety behaviors in nurses.

The results of the present study showed that PSC had a negative relationship with job demands. The association between PSC and job demands has been confirmed in many studies [55–57], and thus was a feature of our hypothesized model, and supported in the final model. Thus, it can be concluded that management can play an important role in reducing job demands by creating an environment of high PSC. Job demands are not necessarily considered negative factors, however when job demands are excessive they will turn into stressors, and, according to job demands-resources theory, present negative consequences [25, 58, 59] one of these negative consequences is reduced safety performance. Thus, PSC is indirectly involved in safety performance through job demands. Accordingly, in an environment with high PSC, management actions can prevent the increase of job demands and subsequently improve safety performance.

The results also showed that PSC has a positive relationship with job resources. There are many resources associated with supporting employees in their work, and in this research it was represented by a latent variable comprised of job rewards, job control, and two forms of social support. These variables have all been identified as key job resources in the literature [26], and the results of previous studies on the relationship between PSC and job resources support this finding [23, 44, 60]. In addition, the results of path analysis showed that among the factors in the model, PSC had the highest impact on job resources. This result seems reasonable because management plays an important role in providing job resources. Management can also help increase social support by creating a supportive environment. Therefore, in an environment with high PSC, management strives to maintain employees’ safety and mental health by providing job resources for them. This perception makes it easier for employees to deal with stressors and job demands and it also reduces negative emotions such as emotional exhaustion and job dissatisfaction. As a result, positive behaviors resulting from job satisfaction such as safety performance increase. Therefore, PSC can also affect safety performance by improving job resources. This assertion was supported in the final model.

The present study confirmed the association of PSC with job satisfaction seen in previous studies [58]. Employees experience a higher level of job satisfaction in an environment where they feel that their mental health is as important as their productivity. Since job satisfaction was found to be directly related to safety performance in the final model, it can be concluded that PSC can also enhance nurses’ safety performance by improving their job satisfaction.

Based on the extant literature [20, 24, 61, 62], another hypothesis of the study was the relationship between PSC and emotional exhaustion. The results of SEM showed that PSC was directly correlated with emotional exhaustion. When PSC is high, it means that management cares about employees’ psychosocial health. Thus, employees feel valued, and this reduces their negative feelings in the workplace. Conversely, the lack of a psychosocial safety climate in the organization can increase employees’ willingness to hide emotions instead of expressing them. This happens when managers do not care about the employees’ mental health and do not pay attention to their concerns. The cumulative effect of these stresses, in the long run, leads to a feeling of emotional exhaustion [19, 23] and consequently can negatively affect their safety performance.

In the hypothesized model, the latent variable job resources was considered as a mediator of the relationship between PSC and safety performance. The results showed that job resources were negatively related to emotional exhaustion, as was confirmed in previous studies [29, 31, 63, 64]. This study also showed that job resources were not directly related to job satisfaction but contrary to our hypothesis, they affected job satisfaction through emotional exhaustion. Gountas and Gountas [30] reported results that conflict with the findings of the present study, however their study was focused on the direct relationship between social support (from colleagues and managers) with job satisfaction. When a broader view of job resources is considered, the findings of most studies supported the results of the present study. Critically, the findings do not support the hypothesized direct association between job resources and safety performance, although job resources was found to be indirectly correlated with safety performance through emotional exhaustion and job satisfaction. Previous studies have reported different results. As an example, Turner et al. showed an association of job resources and safety participation, but no correlation between job resources and safety compliance [65], whilst Guo et al. found that job resources were associated with safety compliance but not significantly with safety participation [66]. In contrast, Bronkhorst reported a significant relationship between supervisor support, coworker support, and job control with safe physical behavior [15]. Ultimately, no definite conclusion can be drawn about the relationship between job resources and safety performance.

There is more consistent evidence for an indirect link between job resources and safety performance, in line with our finding of a new path for the relationship between job resources and safety performance through emotional exhaustion and job satisfaction. No study has addressed this issue yet. Nevertheless, many studies have confirmed the relationship between job resources and emotional exhaustion and the association of emotional exhaustion with job satisfaction and safety performance. Generally, it can be concluded that job resources have the greatest impact on emotional exhaustion and they can affect other factors such as job satisfaction and safety performance by reducing emotional exhaustion. Therefore, in a hospital setting where nurses have a friendly and supportive relationship with managers and colleagues, they have the power to control the performance of tasks, they can share positive and negative feelings and experiences from daily encounters with patients and their families with their colleagues and managers, they receive adequate material and spiritual rewards in return for the efforts they make, and thus it is easier for them to deal with workplace stressors. In such situations, employees have more positive emotions, which improves their mental health and reduces any emotional exhaustion [31, 67].

In addition to the direct impact of job demands on safety performance, job demands were also indirectly related to safety performance through emotional exhaustion and job demands. This finding supported our evidence-based hypothesis [11, 15, 64]. Therefore, given the direct and indirect impacts of job demands on safety performance, job demands can be considered as an important factor in predicting the safety performance of health care and medical staff. Accordingly, it can be argued that employees may ignore safety instructions when faced with high job demands because they spend their time completing their duties. On the other hand, when job demands are high, employees try to complete their tasks instead of taking the time to volunteer to improve safety, and this, in turn, decreases the level of safety performance [65]. Furthermore, job demands indirectly affect safety performance through emotional exhaustion and job satisfaction. According to the path of health disorders, when job demands are not commensurate with employees’ ability, achieving job goals becomes difficult for employees and they will have to spend a lot of energy to complete their jobs. Moreover, if high job demands are persistent, it leads to employees’ energy depletion and ultimately emotional exhaustion [63, 68]. The experience of emotional exhaustion over time will reduce nurses’ satisfaction with their work and work environment and ultimately leads to a decrease in safety performance, indicating intervention is required to prevent this situation.

Emotional exhaustion is known as the main component of burnout. The SEM showed that, contrary to the hypothetical model, job satisfaction had no effect on emotional exhaustion and, conversely, emotional exhaustion was a predictor of job satisfaction and was indirectly related to safety performance. Both paths have been reported in previous studies [63, 69, 70]. However, there has been some controversy regarding whether or not emotional exhaustion is a predictor of job satisfaction [69, 71]. Differences in findings may be due to the use of different measures of job satisfaction. Although previous studies have suggested that emotional exhaustion is a predictor of safety performance, most of these studies that have not found a relationship have used lagging indicators of safety performance such as reported accidents [29] or final safety consequences [29]. On the other hand, studies to date have not examined the relationship between emotional exhaustion and safety performance through job satisfaction, even though the relationship between job satisfaction and safety performance has been confirmed by previous studies [51, 72]. Therefore, one of the contributions of the present study was the introduction of a new path to predicting safety performance.

### Strengths and Limitations

The present study developed a new model that can be used to come up with a better understanding of the factors affecting safety performance behaviors in nurses. This model can support interventions to ameliorate risks for occupational accidents. Specifically, this study incorporated the effects of psychosocial factors such as psychosocial safety climate and psychological factors including emotional exhaustion and job satisfaction as well as job characteristics like job demands and resources to understand safety performance. In most previous studies, investigations of safety performance have been restricted to considering the contribution of physical safety climate and ignore the psychosocial dimensions of safety climate. Nevertheless, the present study showed that psychosocial safety climate was a powerful factor in predicting nurses’ safety performance.

Despite its strengths, the study was conducted with some limitations. For instance, the research sample included nurses in one city in Iran. Nevertheless, we suggest that their job and the context of their work is similar throughout Iran and many parts of the world – particularly at the time of the study during the global pandemic. The data in this study were collected using self-report instruments and the potential for social desirability bias is ever present. In this study, an attempt was made to incorporate the most important organizational and psychosocial factors affecting safety performance in a model. However, given the wide range of factors influencing safety performance, it is impossible to consider all factors in a single study. Therefore, other individual factors, especially employees’ psychosocial and psychological factors must be explored in future studies. Another limitation of the present study that is common in all cross-sectional studies is that they cannot explain cause-and-effect relationships. Moreover, despite the advantages of structural equation modeling, its mathematical foundation is based on the assumption of linear relations. The relationships between psychological and environmental variables are not necessarily linear and can be a function of nonlinear relationships. Therefore, it is suggested to use nonlinear and curved models such as artificial neural networks to better understand the impact of psychosocial factors on safety performance and other variables are used in due course.

## Conclusion

The present study employed SEM to develop a new model for predicting safety performance based on the psychosocial safety climate. The assessment of the model showed that the psychosocial safety climate, both directly and indirectly by affecting all variables in the model from four different paths, can help improve nurses’ safety performance. Therefore, it can be concluded that the psychosocial safety climate plays a very important role in predicting nurses’ safety performance, and in addition to paying attention to the physical aspects of the workplace, organizations should also take into account PSC to improve safety.

## Data Availability

The datasets used and/or analyzed during the current study are available from the corresponding author on reasonable request.

## Abbreviations

PSC: Psychosocial Safety Climate
SEM: Structural Equation Modeling
ERIQ: The Effort-Reward Imbalance Questionnaire
MOAQ-JSS: The Michigan Organizational Assessment Questionnaire Job Satisfaction Subscale
MBI: The Maslach Burnout Inventory
CVR: Content Validity Ratio
CVI: Content Validity Index
CFA: Confirmatory Factor Analysis
χ^2^/df: Chi-Square/Degrees of Freedom Ratio
RMSEA: Root Mean Square Error of Approximation
GFI: Goodness-of-Fit Index
AGFI: Adjusted Goodness-of-Fit Index
CFI: Comparative Fit Index
